# An Intensive Exercise Program Using a Technology-Enriched Rehabilitation Gym for the Recovery of Function in People With Chronic Stroke: Usability Study

**DOI:** 10.2196/46619

**Published:** 2023-07-21

**Authors:** Andy Kerr, Maisie Keogh, Milena Slachetka, Madeleine Grealy, Philip Rowe

**Affiliations:** 1 Department of Biomedical Engineering University of Strathclyde Glasgow United Kingdom; 2 School of Psychological Sciences and Health University of Strathclyde Glasgow United Kingdom

**Keywords:** rehabilitation technology, stroke, feasibility, intensive exercise, rehabilitation, exercise, motor impairment, feasibility study, telehealth, recovery, telerehabilitation

## Abstract

**Background:**

Rehabilitation improves poststroke recovery with greater effect for many when applied intensively within enriched environments. The failure of health care providers to achieve minimum recommendations for rehabilitation motivated the development of a technology-enriched rehabilitation gym (TERG) that enables individuals under supervision to perform high-intensity self-managed exercises safely in an enriched environment.

**Objective:**

This study aimed to assess the feasibility of the TERG approach and gather preliminary evidence of its effect for future research.

**Methods:**

This feasibility study recruited people well enough to exercise but living with motor impairment following a stroke at least 12 months previously. Following assessment, an 8-week exercise program using a TERG (eg, virtual reality treadmills, power-assisted equipment, balance trainers, and upper limb training systems) was structured in partnership with participants. The feasibility was assessed through recruitment, retention, and adherence rates along with participant interviews. Effect sizes were calculated from the mean change in standard outcome measures.

**Results:**

In total, 70 individuals registered interest, the first 50 were invited for assessment, 39 attended, and 31 were eligible and consented. Following a pilot study (n=5), 26 individuals (mean age 60.4, SD 13.3 years; mean 39.0, SD 29.2 months post stroke; n=17 males; n=10 with aphasia) were recruited to a feasibility study, which 25 individuals completed. Participants attended an average of 18.7 (SD 6.2) sessions with an 82% attendance rate. Reasons for nonattendance related to personal life, illness, weather, care, and transport. In total, 19 adverse events were reported: muscle or joint pain, fatigue, dizziness, and viral illness, all resolved within a week. Participants found the TERG program to be a positive experience with the equipment highly usable albeit with some need for individual tailoring to accommodate body shape and impairment. The inclusion of performance feedback and gamification was well received. Mean improvements in outcome measures were recorded across all domains with low to medium effect sizes.

**Conclusions:**

This study assessed the feasibility of a holistic technology-based solution to the gap between stroke rehabilitation recommendations and provision. The results clearly demonstrate a rehabilitation program delivered through a TERG is feasible in terms of recruitment, retention, adherence, and user acceptability and may lead to considerable improvement in function, even in a chronic stroke population.

**International Registered Report Identifier (IRRID):**

RR2-doi.org/10.3389/fresc.2021.820929

## Introduction

Globally, 2.41 billion people live with conditions that can be improved with rehabilitation [1]. As the leading cause of long-term disability, stroke makes up a considerable proportion of this population [2] and is responsible for the loss of an estimated 18 million years to disability [1]. Evidence-based guidelines for delivering the type of rehabilitation known to improve recovery and reduce disability after stroke are widely available [3]. Globally adopted [4,5], these guidelines recommend an approach that is individually tailored, intensive, and delivered within enriched environments. The overwhelming need for this rehabilitation, however, far outstrips the capacity of most health care systems, which are constrained by dependency on specialist staff, resulting in suboptimal, and often inequitable, rehabilitation. The mismatch between what is required and what can be delivered has been repeatedly documented in the United Kingdom [6] and globally [7,8].

As a potential solution to scaling up intensity, technology has gradually been adopted into practice. Rehabilitation technology like treadmills [9], speech therapy apps [10], virtual reality [11], and telerehabilitation have slowly been put into practice. These changes were accelerated by restrictions on face-to-face therapy during the recent COVID-19 pandemic [12]. Despite promising and consistent evidence of effect, the adoption of rehabilitation technology into practice continues to be patchy without real adjustment to the underlying labor-intensive delivery model [13]. Furthermore, when technology has been trialed, it has typically been done in isolation and not part of a holistic, integrated intervention; an approach considered critical for complex health challenges [14].

Our multidisciplinary rehabilitation research group at the University of Strathclyde (Glasgow, UK) has established a cocreation center for rehabilitation technology [15]. The center offers an 8-week rehabilitation program located in a gym-like space equipped with a range of integrated technology designed to holistically address the motor and communication impairments caused by stroke. Further details of this technology-enriched rehabilitation gym (TERG) can be found in our previous publication [15]. The center and program are supervised by trained staff on a one-to-many basis with individuals encouraged to define their rehabilitation goals and, with support, manage their program.

The aim of this study was to assess whether this supported self-managed approach undertaken in a TERG was feasible and acceptable to a group of chronic stroke survivors and to collect data that would allow an effect size to be estimated for future research.

## Methods

### Overview

Details of the methods, including participant eligibility, rehabilitation equipment, and example programs, are available in our previous publication [15]. Here, we describe the key elements of the methods according to the CONSORT (Consolidated Standards of Reporting Trials) guideline extension for reporting pilot and feasibility studies [16]; the checklist is provided (omitting the randomization protocol) in Multimedia Appendix 1.

### Ethics Approval

This study was approved by the University of Strathclyde ethics board (UEC20/08).

### Design

This is a feasibility study of a novel, technology-based, rehabilitation intervention in a group of chronic stroke survivors. Feasibility was assessed through recruitment, retention, attendance, and adherence to the program, safety (incidence and nature of adverse and serious adverse events [AEs]), and participant acceptability using a mixed methods approach including semistructured interviews, attendance, activity, and safety records.

### Participants

People living with stroke affecting their mobility or communication but otherwise well enough for light or moderate exercise were invited to participate. Recruitment was through a network run by a medical charity for stroke in Scotland. Individuals expressing an interest in participating registered with the charity and were invited, in the order they registered, to attend an initial meeting where eligibility was assessed and baseline measures of function recorded.

### Intervention Details

The intervention was developed from our previous work [15,17-19] and feedback from a pilot with chronic stroke survivors (n=5; mean age 51.6, SD 12.1 years; mean 19.6, SD 9.32 months post stroke; 2 females; 2 with aphasia). The small pilot sample size and limited attendance (twice weekly) were related to COVID-19 restrictions in place at the time. Feedback from these participants, through independent interviews, allowed us to implement changes to the intervention, most importantly this included an increase in the number of weekly available sessions from 2 to 5.

The resulting 8-week long rehabilitation intervention was delivered entirely through technology, including virtual reality (immersed and nonimmersed), treadmills, weight suspension and movement resistance, and assistance equipment located in a gym-like space on a university campus (Glasgow, UK). Individual programs were designed, supervised, and reviewed by a physiotherapist using principles of intensity, feedback, cognitive engagement, and aerobic activity [20] to address the goals identified by the participant and scores from outcome measures at baseline. An example program is detailed in a previous publication [15]. Participants were encouraged to use the exercise equipment on their own, wherever possible, while being supervised and to make alterations to the program, with support from the therapist.

### Outcome Measures

Feasibility was assessed by rates of recruitment, adherence, AEs, and participants’ perceptions of acceptability from semistructured interviews [21] (see Multimedia Appendix 2 for interview schedule). To reflect the multidomain nature of the intervention, a range of outcome measures were included: 10-meter walk test (10mWT), five times sit to stand test, action research arm test, functional ambulatory category, Rivermead Mobility Index, and the Stroke Impact Scale-16 (SIS-16) [22-26].

### Data Analysis

Participant interviews were analyzed using the 6-stage thematic approach described by Braun and Clarke [27]. Initially, an independent researcher generated codes and candidate themes. These were then reviewed by 2 members of the research team. Through an iterative process of discussing and revising, a consensus was reached. Descriptive statistics were used to assess feasibility (recruitment, retention, adherence, and safety) and outcome data.

## Results

### Recruitment

Between August 2021 and August 2022, 70 individuals registered their interest in participating in this study. The first 50 were invited to attend an initial meeting to assess eligibility, 39 attended, and 31 met the criteria and consented. In total, 8 individuals were not eligible due to conflict with ongoing rehabilitation (n=2), currently unwell or in pain (n=2), unable to attend at least twice a week due to lack of transport (n=2) or other reasons (n=1), and other (n=1). The first 5 recruited participants participated in a pilot of the intervention with the next 26 participating in the feasibility study. A participant flowchart is available in Multimedia Appendix 3. Since the program continues to be supported through charitable funding, the 20 individuals still on the register will be invited to participate in future cohorts. Full details of the sample, separated into 3-phased participating cohorts, recruited to this feasibility study are provided in Table 1.

**Table 1:** Participant details separated into the 3 cohorts.

	Age (years), mean (SD)	Gender (female/male), n	Time since stroke (months), mean (SD)	Aphasia, n	MoCA^a^, mean (SD)	Attendance, mean number of sessions (SD)
Cohort 1 (n=9)^b^	57.4 (17.7)	3/6	51.1 (34.8)	2	26.7 (2.1)	15.4 (3.3)
Cohort 2 (n=7)	61.9 (12.9)	4/3	20.9 (17.3)	4	21.17 (8.6)	20.1 (0.8)
Cohort 3 (n=10)	62 (9.1)	3/7	42.6 (26.6)	4	21.2 (9.8)	21.3 (6.9)
Total (N=26)	60.4 (13.3)	9/17	39.0 (2.2)	10	23.1 (8.3)	18.7 (4.9)

^a^MoCA: Montreal Cognitive Assessment.

^b^One participant withdrew completely from this group after 3 weeks, citing a lack of transport.

### Program Adherence

All participants set individual goals in partnership with a physiotherapist, including the number of weekly sessions. A total of 493 total sessions were attended representing 986 hours of therapy. In total, 5 individuals achieved, or exceeded, their target number of sessions, and there was, overall, an average adherence rate of 82% (number of attended sessions or number of sessions planned). In total, 21 participants missed a total of 91 (18% of total) planned sessions for the following reasons: illness (n=13), hospital appointment (n=4), weather (n=15), work (n=5), vaccination (n=5), holidays (n=8), personal (n=24), child care (n=7), and transport (n=12).

### Safety

No serious AEs were reported during this study. There were, however, a number of AEs reported (n=19) considered to be related to the study: joint or muscle soreness (n=6), viral illness (including COVID-19; n=5), cardiovascular (dizziness; n=3), fatigue (n=3), and skin irritation (n=2). These all resolved within 1 week without intervention.

### Semistructured Interviews

#### Overview

Participants from cohorts 1 and 3 (n=19) were invited to be interviewed remotely by a researcher not directly involved in the delivery of the therapy after their participation. In total, 12 (63%) individuals agreed. The interviews explored the acceptability of the intervention including the usability of the equipment, perceptions of technology-based feedback, and the need for supervision. Participants were also asked if they achieved their overall goal and whether they perceived any changes in their quality of life. The potential for home use, future plans, and areas for improvement were also explored. Six themes emerged from the analysis.

#### Equipment Usability

The majority of interviewed participants (9/12) found all the equipment used in the TERG to be easy to use with 1 participant commenting “there was nothing I particularly struggled with.” There was some variability in usability, for example, the Shapemaster power-assisted rowing machine, GripAble, Motek Medical “Cube,” and “Functional Squat” were identified as being “highly useable.” The majority of participants (7/12) did find some activities challenging, for example, P15 stated, “It was all quite difficult for me, I think it had to do where you are in stroke journey but everything was useful, it pushed you further. I enjoyed it.” Difficulty in customizing equipment was highlighted as a specific problem by some individuals, particularly in the use of standard grip sizes, which could not always accommodate the range of hand spans and degree of spasticity.

#### Movement Feedback and Gamification

The majority of participants (9/12) valued the use of games and performance feedback provided during the exercises by the technology, with the feedback provided by the equipment to being both helpful (“the treadmill was very innovative, especially with video where I could see myself” [P7]) and motivating (“because it gave a figure to try and better next time” [P4]). The game-based feedback was not, however, universally approved with some participants finding the games a “distraction” and not fully understanding the meaning of the feedback, in particular, how it related to their impairment.

#### Goal Achievement

Half the participants (6/12) felt that they had achieved their overall goal, but for 2 participants, this related to initial goals being too ambitious: “I think there was a degree of progress, maybe not as much as I had hoped but there was progress” (P4) and “I wasn’t expecting to achieve my goals, but it has improved my balance and I can walk faster for longer and with more confidence” (P3). Confidence improved for 8 out of 12 participants, for example, “confidence was improved and external gyms now seem like something that I could try” (P8).

#### Need for Professional Supervision

Almost all participants (11/12) indicated the need for supervisory support for safety and guidance with the equipment and felt the presence of a trained rehabilitation professional to be valuable. This was particularly the case during the treadmill training as this was an area of focused attention for many participants. Two participants voiced a desire to have support from staff reduced over the time of the program to nurture greater independence in the use of the equipment.

#### Potential for Home or Community Use

In total, 11 of 12 participants thought the smaller pieces of equipment (eg, GripAble and Neuroball) were good candidates for home-based rehabilitation. The larger pieces of gym equipment (treadmill and resistance training equipment) were considered to be potentially useful if available in local leisure centers. In total, 11 of 12 participants planned to continue with activity-based rehabilitation.

The smaller pieces of equipment (eg, GripAble and Neuroball) were seen as good candidates for home-based rehabilitation. The larger pieces of gym equipment (treadmill and resistance training equipment) were considered to be potentially useful if available in local leisure centers but with participants being more varied in their confidence using them, given issues of balance and need for support getting in and out of some systems. Participants were also wary of specialist equipment being available for general public use as this would have an impact on availability. Participants also expressed a desire to be independent when using gym equipment.

#### Overall Impression and Rehabilitation Continuation

All participants considered the TERG to have made a very positive impact on their recovery and an experience that helped restore confidence in their physical abilities:

every stroke survivor needs this [TERG] in their life as it was just so positive.

P2

All interviewed participants expressed clear plans to continue their rehabilitation, including home-based work, purchasing rehabilitation equipment, walking outside more, using fitness trackers for motivation, and joining a local gym. While cost issues were highlighted as a potential barrier to these plans, most of the participants (8/12) felt more confident in their physical abilities and were motivated to continue the progress they had made. One participant commented on the potentially negative psychological effect of the program ending, expressing a need for an individual continuation plan.

### Outcome Measures

All participants were able to complete the measurements taken before and after the program. Consistent with previous studies [28], there was considerable variation in group characteristics. In general, there was a positive effect on scores of physical ability with all outcome measures showing a mean improvement (Table 2). Differences were, however, not tested for statistical probability since this study was not set up for this reason; instead, they are reported here as 95% CI and effect size to allow sample size estimations for future studies.

**Table 2:** Mean of outcome measures before and after the program, mean difference, and effect size.

	RMI^a^	SIS-16^b^	FAC^c^	FTSTST^d^	10mWT^e^	ARAT^f^
Before the program, mean (95% CI)	11.0 (9.9 to 12.2)	61.2 (57.9 to 65.6)	3.8 (3.4 to 4.3)	26.8 (17.0 to 36.6)	30.4 (16.6 to 44.2)	29.8 (20.1 to 39.6)
After the program, mean (95% CI)	12.7 (11.9 to 13.6)	66.5 (63.1 to 69.9)	4.5 (4.2 to 4.8)	21.7 (15.5 to 27.8)	21.4 (14.1 to 28.7)	30.8 (20.4 to 41.2)
Difference, mean (95% CI)	1.9 (1.3 to 2.6)	5.5 (3.5 to 7.5)	0.7 (0.4 to 1.0)	−8.0 (−15.4 to −0.6)	−10.6 (−19.4 to −1.7)	3.1 (0.9 to 5.3)
Effect size (Cohen *d*)	0.74	0.60	0.66	−0.41	−0.38	0.13

^a^RMI: Rivermead Mobility Index.

^b^SIS-16: Stroke Impact Scale-16.

^c^FAC: functional ambulatory category.

^d^FTSTST: five times sit to stand test.

^e^10mWT: 10-meter walk test.

^f^ARAT: action research arm test.

## Discussion

### Principal Findings

This study assessed the feasibility of a novel model of stroke rehabilitation designed to deliver evidence-based stroke rehabilitation through the scalable model of a TERG and a self-management, supervised, approach.

### Feasibility

The participant’s variability in age (SD 13.3 years), cognition (Montreal Cognitive Assessment: SD 23.1), communication (n=10, 38% aphasic), severity of motor impairment (action research arm test: SD 23.1 and 10mWT: SD 34.1), and overall impact on their lives (SIS-16: SD 9.6) reflect both the heterogeneity of this population [29] and the broad inclusion criteria. The findings can therefore be applied to the general stroke population with some confidence, albeit with the limitation that participants needed to be medically well, a criterion that excluded 2 potential individuals.

The intervention can be considered feasible within this highly variable population. Program adherence was generally good at 82%, with an average attendance of 2.4 sessions per week, and only 1 participant dropping out completely for transport reasons. Adherence to rehabilitation programs, in general, is low, ranging from 40% to 71% [30] but may be higher among stroke populations when offered in a structured manner, for example, through exercise facilities such as gyms (eg, Reynolds et al [31] report 81% adherence) or when technology is included, Valenzuela et al [32] reported 91% adherence to a technology-based exercise program in older adults. The number of AEs could be considered high (11 participants reporting 19 AEs) but should be seen in the context of the 493 total sessions attended (986 hours), 1 AE every 51 hours, and the minor nature of the AEs, many of which related to joint and muscle discomfort that could be explained by an increase in exercise and the viral illnesses, which should be seen in the context of the contemporaneous COVID-19 pandemic. AEs are relatively common in the poststroke population, Ostwald et al [33] reported 50% of 159 patients tracked after stroke experienced at least 1 AE in the first year poststroke.

Recruitment for this study was managed by a partner organization and considered broadly successful without the need to specifically advertise or promote the center. In trying to achieve 10 people for each group, 11 individuals were targeted for the assessment sessions (allowing for 10% attrition); however, across all the groups, 8 potential recruits were deemed ineligible due to an inability to attend frequently enough (transport and other reasons) or current pain or illness that prohibited use of the equipment. This finding suggests 2 improvements for future studies: clearer information at the start of the process (when registering interest) and an increase in the number of people invited to the baseline assessments to ensure that 10 participants start the program.

### Barriers to Attendance

These positive findings of feasibility are balanced against continued reports of barriers to access. Although daily attendance was possible (40 sessions available in total) and encouraged, no participant achieved this; 33 was the highest number of sessions attended by a single participant. While unmodifiable barriers (illness, weather, national holidays, and personal) account for at least some of the issues around fully accessing the program, the lack of transport was mentioned frequently and was also reported in the interviews. This is consistent with previous reports of environmental barriers (including transport) to physical activity in stroke populations [34]. The TERG was situated in a city center campus which, for some, meant relatively long and costly travel arrangements that likely limited participation. Our plans to establish the TERG model in community locations could resolve some of these difficulties and have been strongly recommended by the World Health Organization [35].

Motor impairments have previously been reported as barriers to using equipment for exercise or physical activity participation [36]. Reassuringly, in this study, this was only mentioned in relation to grip, suggesting the existing adaptations to the TERG equipment enabled broad participation.

### Changes in Outcome Measures

While this study was not designed to test efficacy, positive change to all outcome measures is worth noting, in particular, the changes to gait (mean reduction of 10.6 s in 10mWT) and sit to stand ability (mean reduction of 8 s in the five times sit to stand test) and the moderate effect sizes (0.74 and 0.60) estimated for Rivermead Mobility Index and SIS-16, respectively. The high variability in the change data (eg, the SD for change in 10mWT was 27.5 s) further demonstrates the variability of response to rehabilitation in this population that merits further investigation to understand explanatory factors. Despite this variability, improvements compare well to rehabilitation interventions in stroke [37] and suggest that greater improvements may be possible during the subacute phase and that time since stroke should not be seen as an exclusion factor to this kind of rehabilitation program.

### Limitations

A number of limitations should be considered when interpreting these findings. In particular, the lack of a comparator group means that any change recorded in physical ability may relate to natural recovery or a Hawthorne effect [37] and not the intervention. The chronic nature of the participants, however, suggests natural recovery is likely to be a small part of the positive response.

A greater issue, for interpreting feasibility and effect size, is the recruitment process, which is likely to be biased toward individuals with a pre-existing motivation and interest in rehabilitation. While this cannot be avoided in the context of research or ethics governance, it may mean that metrics like recruitment, retention, and adherence may not be as positive in the real world.

No cost analysis was performed on the intervention. This is recommended for future studies but should include health and societal benefits, including a return to economic and social activity, where appropriate.

### Recommendations

Based on the findings of this feasibility study, a number of recommendations are suggested for further study and development: (1) establishing community versions of the TERG to resolve access barriers, (2) statistically powered randomized controlled trial of efficacy, (3) health economics analysis of the intervention, and (4) adaptable gripping systems for exercise equipment.

### Conclusions

A novel approach to stroke rehabilitation using a TERG with professional supervision is feasible, with 82% attendance across almost 1000 hours of delivery and with only minor AEs reported. Reassuringly, the intervention was overwhelmingly well received by this diverse group of chronic stroke survivors. This approach has the potential to meet the overwhelming need for greater access to effective rehabilitation but requires an experimental approach, with a statistically powered sample, to confirm the early promising findings.

## Appendix

Multimedia Appendix 1 CONSORT (Consolidated Standards of Reporting Trials) extension for pilot and feasibility reporting.

Multimedia Appendix 2 Interview schedule.

Multimedia Appendix 3 Recruitment flowchart.

## Abbreviations

10mWT: 10-meter walk test
AE: adverse event
CONSORT: Consolidated Standards of Reporting Trials
SIS-16: Stroke Impact Scale-16
TERG: technology-enriched rehabilitation gym
