# Supplementary material for: An Intensive Exercise Program Using a Technology-Enriched Rehabilitation Gym for the Recovery of Function in People With Chronic Stroke: Usability Study
Source: JMIR Rehabil Assist Technol. 2023 Jul 21;10:e46619. doi: 10.2196/46619 (PMC10403794; doi:10.2196/46619)
Supplement: Multimedia Appendix 1 [file rehab_v10i1e46619_app1.pdf]

| Section/topic and item No | Extension for pilot trials | Page No where item is reported |
|---|---|---|
| Title and abstract | | |
| 1a | Identification as a pilot or feasibility randomised trial in the title | 2 |
| 1b | Structured summary of pilot trial design, methods, results, and conclusions (for specific guidance see CONSORT abstract extension for pilot trials) | 2 |
| Introduction | | |
| Background and objectives: | | |
| 2a | Scientific background and explanation of rationale for future definitive trial, and reasons for randomised pilot trial | 3 |
| 2b | Specific objectives or research questions for pilot trial | 3 |
| Methods | | |
| Trial design: | | |
| 3a | Description of pilot trial design (such as parallel, factorial) including allocation ratio | 3 |
| 3b | Important changes to methods after pilot trial commencement (such as eligibility criteria), with reasons | 4 |
| Participants: | | |
| 4a | Eligibility criteria for participants | 4 |
| 4b | Settings and locations where the data were collected | 4 |
| 4c | How participants were identified and consented | 4 |
| Interventions: | | |
| 5 | The interventions for each group with sufficient details to allow replication, including how and when they were actually administered | 4 |
| Outcomes: | | |

| | | |
|---|---|---|
| 6a | Completely defined prespecified assessments or measurements to address each pilot trial objective specified in 2b, including how and when they were assessed | |
| 6b | Any changes to pilot trial assessments or measurements after the pilot trial commenced, with reasons | 4 |
| 6c | If applicable, prespecified criteria used to judge whether, or how, to proceed with future definitive trial | |
| Sample size: | | |
| 7a | Rationale for numbers in the pilot trial | |
| 7b | When applicable, explanation of any interim analyses and stopping guidelines | |
| Randomisation: | Omitted sections 8-11 | |
| Analytical methods | | |
| 12 | Methods used to address each pilot trial objective whether qualitative or quantitative | 4 |
| Results | | |
| 13a | For each group, the numbers of participants who were approached and/or assessed for eligibility, randomly assigned, received intended treatment, and were assessed for each objective | 4 and supplementary |
| Recruitment: | | |
| 14a | Dates defining the periods of recruitment and follow-up | 4 |
| 14b | Why the pilot trial ended or was stopped | |
| Baseline data: | | |
| 15 | A table showing baseline demographic and clinical characteristics for each group | 5 |
| Numbers analysed: | | |
| 16 | For each objective, number of participants (denominator) included in each analysis. If relevant, these numbers should be by randomised group | |

| | | |
|---|---|---|
| Outcomes and estimation | | |
| 17 | For each objective, results including expressions of uncertainty (such as 95% confidence interval) for any estimates. If relevant, these results should be by randomised group | 7 |
| Ancillary analyses: | | |
| 18 | Results of any other analyses performed that could be used to inform the future definitive tria | 7 |
| Harms: | | |
| 19a | All important harms or unintended effects in each group (for specific guidance see CONSORT for harms) | 5 |
| 19b | If relevant, other important unintended consequences | |
| Discussion | | |
| 20 | Pilot trial limitations, addressing sources of potential bias and remaining uncertainty about feasibility | 7,8 |
| Generalisability: | | |
| 21 | Generalisability (applicability) of pilot trial methods and findings to future definitive trial and other studies | |
| Interpretation: | | |
| 22a | Interpretation consistent with pilot trial objectives and findings, balancing potential benefits and harms, and considering other relevant evidence | 7,8 |
| 22b | Implications for progression from pilot to future definitive trial, including any proposed amendments | 8 |
| Other information | | |
| Registration: | Registration number for pilot trial and name of trial registry | |
| Protocol: | | |
| 24 | Where the pilot trial protocol can be accessed, if available | 3 |
| Funding: | | |

| | | |
|---|---|---|
| 25 | Sources of funding and other support (such as supply of drugs), role of funders | 9 |
| 26 | Ethical approval or approval by research review committee, confirmed with reference number | 3 |
