# Supplementary material for: An Intensive Exercise Program Using a Technology-Enriched Rehabilitation Gym for the Recovery of Function in People With Chronic Stroke: Usability Study
Source: JMIR Rehabil Assist Technol. 2023 Jul 21;10:e46619. doi: 10.2196/46619 (PMC10403794; doi:10.2196/46619)
Supplement: Multimedia Appendix 2 [file rehab_v10i1e46619_app2.doc]

Semi-structured interview

Participants were informed that the interview would last approximately 30 minutes. The interviewer will be an individual who was not involved in the delivery of the rehabilitation and will be conducted in a quiet private meeting room.

The interview started with setting out the reasons for collecting this information (i.e. to improve design and translation of rehabilitation technologies into routine practice). The following areas will be discussed. Questions are indicative.

**Usability of the equipment**

- How did you find the treadmill/bike/balance trainer/arm trainer/speech app?
- Was there anything you found particularly difficult to manage?
- Was there anything you found particularly easy to manage?

**Perceptions of technology based feedback**

- Did you find the progress information provided by the equipment useful?
- Were you able to access the feedback on the equipment easily enough?
- Did you need help understanding it?

**Need for supervision**

- How important do you think it was to have someone present during your sessions?
- Do you think you needed a trained person present?
- Was there any equipment you felt you could manage without help?

**Goals and changes**

- Did you feel you achieve your overall goal at the end of the programme?
- Do you think there have been any changes in your speech/mobility/balance/confidence/quality of life?
- Was there any particular piece of equipment that you think helped more?

**Potential for home use**

- Would you consider using any of the equipment at home or at a local leisure centre?
- Would you anticipate any difficulties using this equipment at home?
- What support do you think you might need/like to use this equipment at home?

**Future plans**

- Now that you have completed the rehabilitation programme will you continue any activities/rehabilitation exercises?
- Do you have any plans to continue with rehabilitation?

**Suggestions for improvement**

- Now that you have experienced rehabilitation using machines and equipment do you have any suggestions for improvement?
- Thinking about the specific equipment you used do you think we could improve it?
- Do you have any ideas how else we might help people in the programme?
- How do you think we could have improved the overall experience for you?
