# Supplementary figures and images for: An Intensive Exercise Program Using a Technology-Enriched Rehabilitation Gym for the Recovery of Function in People With Chronic Stroke: Usability Study
Source: JMIR Rehabil Assist Technol. 2023 Jul 21;10:e46619. doi: 10.2196/46619 (PMC10403794; doi:10.2196/46619)

## Recruitment flowchart

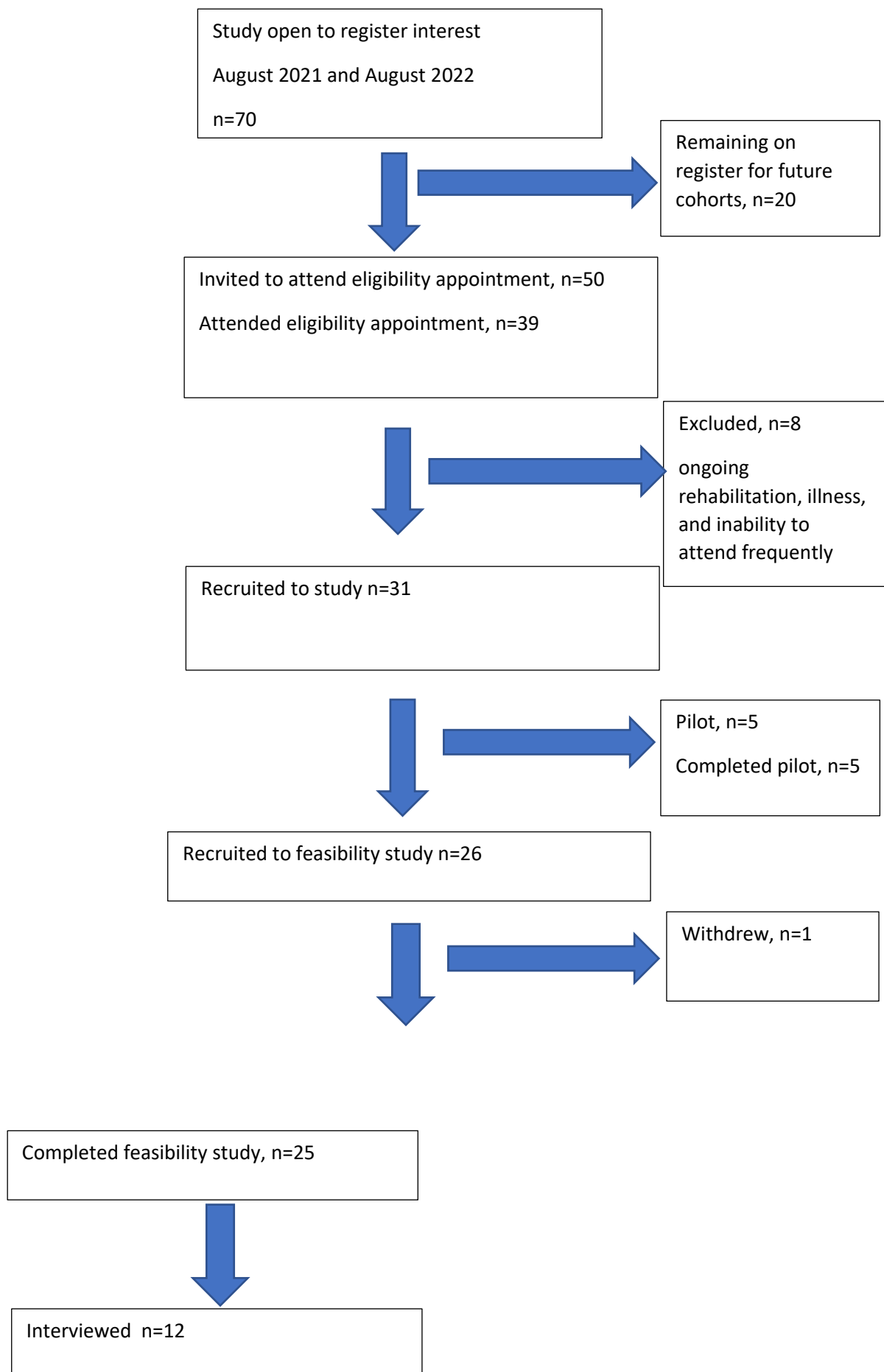

Supplement: Multimedia Appendix 3 [file rehab_v10i1e46619_app3.pdf]
